# Down‐regulation of endothelial protein C receptor promotes preeclampsia by affecting actin polymerization

**DOI:** 10.1111/jcmm.15011

**Published:** 2020-01-30

**Authors:** Hao Wang, Pan Wang, Xiaoling Liang, Wenjing Li, Mo Yang, Jihong Ma, Wei Yue, Shangrong Fan

**Affiliations:** ^1^ Department of Obstetrics and Gynecology Peking University Shenzhen Hospital Shenzhen China; ^2^ Shenzhen Key Laboratory on Technology for Early Diagnosis of Major Gynecological Diseases Shenzhen China; ^3^ Department of Obstetrics and Gynecology Peking University Third Hospital Beijing China; ^4^ Department of Obstetrics and Gynecology Sir Run Run Shaw Hospital Zhejiang University School of Medicine Hangzhou China; ^5^ Medical Center for Human Reproduction Beijing Chaoyang Hospital Capital Medical University Beijing China

**Keywords:** cell invasion, cell proliferation, endothelial protein C receptor, preeclampsia, tube formation

## Abstract

Preeclampsia is a severe pregnancy‐related disease that is found in 3%–5% of pregnancies worldwide and is primarily related to the decreased proliferation and invasion of trophoblast cells and abnormal uterine spiral artery remodelling. However, studies on the pathogenesis of placental trophoblasts are insufficient, and the aetiology of PE remains unclear. Here, we report that endothelial protein C receptor (EPCR), a transmembrane glycoprotein, was down‐regulated in placentas from preeclamptic patients. Moreover, lack of EPCR significantly reduced the trophoblast cell proliferation, invasion and tube formation capabilities. Microscale thermophoresis analysis showed that EPCR directly bound to protease‐activated receptor 1 (PAR‐1), a G protein‐coupled receptor. This change resulted in a substantial reduction in active Rac1 and caused excessive actin rearrangement. Our findings reveal a previously unidentified role of EPCR in the regulation of trophoblast proliferation, invasion and tube formation through promotion of actin polymerization, which is required for normal placental development.

## 1 INTRODUCTION

Preeclampsia (PE) is a complex, pregnancy‐specific disease characterized by the development of hypertension and proteinuria after 20 weeks of gestation.^1^ This condition is one of the major causes of maternal, perinatal and foetal/neonatal mortality and morbidity worldwide.^2^, ^3^ To date, the aetiology of PE has remained elusive, but we know that placental pathology is involved and plays a crucial role. The most significant pathological change of PE is believed to be impaired extravillous trophoblast (EVT) proliferation and invasion accompanied by poor spiral vascular remodelling.^4^ Therefore, the volume of maternal blood flowing into the intervillous space is decreased, which causes several negative consequences, including high pressure, abnormal stress in the villous placenta and increased shedding of necrotic placental debris in the maternal circulation.^5^, ^6^ Many cell characteristics, such as cell shape, cell proliferation and invasion, are directly determined by actin.^7^, ^8^ Moreover, column cytotrophoblasts that differentiate into EVTs are related to changes in cell behaviour and cytoskeletal organization,^9^, ^10^ suggesting critical roles for the integrity and appropriate remodelling of the cell cytoskeleton in trophoblast proliferation and invasion.

Endothelial protein C receptor (EPCR), a 46‐kDa transmembrane glycoprotein, is highly expressed in trophoblasts compared to other parts of the maternal‐foetal interface during pregnancy,^11^ and EPCR expression is down‐regulated in preeclamptic placentas compared with healthy placentas.^12^ The sequence and structure of EPCR is homologous to that of proteins of the major histocompatibility class 1/cluster of differentiation 1 (CD1) family,^13^ and this protein participates in many cytoprotective activities, reducing the damage caused by diverse injuries or diseases from sepsis to stroke.^7^ EPCR was reported to participate in the activation of endogenous protease‐activated receptor 1 (PAR‐1), a G protein‐coupled receptor that plays an important role in mediating anti‐inflammatory and antiapoptotic activities and protects endothelial barrier functions through regulating cytoskeletal organization.^14^ Recently, cancer research has shown that EPCR aberrations are involved in diverse tumour tissues, such as breast cancer tissues,^15^ lung cancer tissues^16^, ^17^ and ovarian cancer tissues.^18^ Overexpression of EPCR is associated with the promotion of tumour growth and infiltration. Many studies have demonstrated striking similarities in the molecular circuits between trophoblasts and cancer cells due to the properties of proliferation and invasion of these two cell types.^19^, ^20^ However, whether EPCR is associated with trophoblast function and PE occurrence and development remains unclear. Therefore, we hypothesized that in trophoblast cells, EPCR may participate in the development of PE by affecting cell proliferation and infiltration.

In the current study, we detected EPCR down‐regulation in the placentas of patients with PE compared to those from healthy controls. EPCR knockout inhibited cell proliferation, invasion and tube formation. Microscale thermophoresis (MST) analysis showed that EPCR directly bound to PAR‐1. The decrease in cleaved PAR‐1 caused by EPCR depletion resulted in a substantial reduction in active Rac1, a key factor that regulates the polymerization and depolymerization of actin; these changes resulted in excessive rearrangement of the cytoskeleton. In brief, our study revealed that EPCR down‐regulation resulted in decreased proliferation, invasion and tube formation via the rearrangement of actin through the PAR‐1/Rac1 signalling pathway in placental trophoblasts in PE.

## 2 MATERIALS AND METHODS

### 2.1 Collection of placental tissues

The protocols for placental tissue collection were reported previously.^21^ Placental tissues were obtained from PE patients (n = 15) and subjects with healthy pregnancies (n = 15) admitted to Shenzhen Hospital, Peking University. PE was diagnosed by a new onset of systolic blood pressure ≥ 140 mmHg or diastolic blood pressure ≥ 90 mmHg after the 20th week of gestation in the presence of proteinuria without preexisting renal diseases or primary hypertension. Patients with other major pregnancy complications were excluded. First‐trimester villous tissue (n = 5) was collected from women who underwent legal termination between 6 and 10 weeks of gestational age that was not for medical reasons; these subjects did not have a history of a spontaneous abortion or an ectopic pregnancy. All placental tissues were collected according to protocols approved by the Research Ethics Committee of Shenzhen Hospital, Peking University. Written informed consent was obtained from all patients. The clinical features of the patients are displayed in Table 1.

**Table 1:** Clinical parameters of the control and preeclamptic women recruited for the study

	Control (n = 15)	Preeclampsia (n = 15)	*P*‐values
Maternal ages	29.11 ± 1.567	32.33 ± 1.19	.1211
BMI at delivery (kg/m^2^)	27.78 ± 1.718	30.84 ± 1.381	.1838
Gestational age at delivery (weeks)	38.11 ± 0.389	37.33 ± 0.333	.1484
Systolic BP (mm Hg)	115.7 ± 2.16	153.9 ± 4.296	<.0001
Diastolic BP (mm Hg)	75.11 ± 1.996	98.78 ± 2.639	<.0001
Proteinuria	–	+ to +++	<.0001
Proteinuria (g/24 h)	0 ± 0	2.94 ± 1.102	.0157
Infant birth weight (g)	3328 ± 167.1	2739 ± 233.3	.0569

Note

Values are the mean ± SD.

Abbreviations: BMI, body mass index (in kg/m^2^); BP, blood pressure.

### 2.2 Immunohistochemistry

The villous and placental tissues were washed with phosphate‐buffered saline (PBS) and fixed overnight with 4% paraformaldehyde (PFA) at room temperature. Then, the samples were dehydrated and embedded in paraffin before they were sectioned into 4‐mm‐thick sections. For Immunohistochemistry (IHC), the sections were deparaffinized, rehydrated and then microwaved in 10 mmol/L citric sodium (pH 6.0) for 20 minutes to retrieve antigens and blocked with 3% H_2_O_2_ for 10 minutes. The sections were then incubated with a mouse primary antibody against EPCR (1:200; Abcam, Cambridge, United Kingdom) at 4°C overnight. Then, a secondary antibody conjugated to horseradish peroxidase (HRP) was applied for 20 minutes at room temperature, followed by development in diaminobenzidine solution.

### 2.3 Western blot analysis

Protein extracts were prepared from placental tissues and cells with RIPA buffer supplemented with protease and phosphatase inhibitors. For western blot (WB) analysis, the cell lysates (40 μg of total protein) were electrophoresed by 10% SDS‐PAGE and electrically transferred to a hydrophobic polyvinylidene difluoride membrane (Hybond‐P; Amersham Biosciences, Piscataway, NJ, USA). Following transfer, the membrane was blocked in 5% milk in TBST (0.15 M NaCl, 0.01 M Tris‐HCl and 0.1% Tween‐20, pH 7.4) for 1 hour and extensively washed with TBST. Then, the membrane was incubated with primary antibodies (anti‐EPCR, Abcam; anti‐Rac1, Cytoskeleton, Inc, Danvers, MA, USA; anti‐actin, Cytoskeleton; anti‐GAPDH, Abcam) overnight at 4°C. After three washes in TBST, the membrane was incubated with an appropriate HRP‐conjugated secondary antibody for 1 hour at room temperature. Finally, the membranes were detected with an enhanced chemiluminescence detection system (Amersham Biosciences, Piscataway, NJ, USA).

### 2.4 Cell culture

HTR8/SVneo cells (ATCC No. CRL‐3271) were cultured in phenol red RPMI 1640 medium (Thermo Fisher Scientific, Waltham, MA, USA). The cells were grown at 37°C in 5% CO_2_. All cultures were supplemented with 10% foetal bovine serum (FBS, Thermo Fisher Scientific) and 100 U/ml penicillin‐streptomycin (Thermo Fisher Scientific).

### 2.5 Single‐guide RNA (sgRNA) design, plasmid construction and transfection

sgRNA sequences for the CRISPR/Cas9 system were designed at the CRISPR design website (http://crispor.tefor.net/crispor.py). The sequence of the insert oligonucleotides for the human EPCR gRNA was 5′‐ CCAGGGCAACGCGTCGCTGG‐3′. The EPCR gRNA targets exon 2 of the gene. The complementary oligonucleotides for the gRNAs were cloned into the lentiCRISPRv2 vector (Addgene, Cambridge, MA, USA). For target protein expression in HTR8/SVneo cells, the indicated gene was cloned into pcDNA3. HTR8/SVneo cell lines were transfected with Lipofectamine 3000 transfection reagent (Thermo Fisher Scientific) according to the manufacturer's instructions. After 48 hours of transfection, the expression of EPCR was verified using immunofluorescence (IF) and WB analyses, and subsequent experiments were performed.

### 2.6 Generation of the EPCR knockout (KO) cell line with CRISPR/Cas9

Two days after lentiCRISPRv2 vector transfection, the cells were treated with 2 μg/mL puromycin (Sigma, St. Louis, MO, USA) for three days, and a single cell was isolated. After two weeks, single‐cell colonies were picked, and the EPCR knockout was confirmed by IF staining and WB analysis.

### 2.7 Sequencing

Regions surrounding sgRNA target/off‐target sites within the EPCR were amplified by PCR and analysed by BGI (Shenzhen, China) using Sanger sequencing (ACGT, Inc)

### 2.8 IF staining and confocal microscopy

Cells were fixed in 4% PFA in PBS (pH 7.4) for 30 minutes at room temperature. Then, the cells were permeabilized in 0.5% Triton X‐100 for 30 minutes, followed by blocking for 30 minutes in 1% bovine serum albumin (BSA, Sigma) solution. The cells were then incubated overnight with primary antibodies (1:100‐1:200) at 4°C. After the cells were washed three times in PBS containing 0.1% Tween‐20 and 0.01% Triton X‐100, they were transferred to an appropriate fluorescent secondary antibody mixture for 1 hour at room temperature. The nuclei were then stained with 10 μmol/L DAPI for 10 minutes and washed three times. Finally, the cells were mounted onto glass slides. Observation was performed under a confocal laser scanning microscope (Carl Zeiss 710, Jena, Germany).

### 2.9 Three‑dimensional (3D) modelling

Actin was imaged as a z‐series at 1 µm intervals to capture the entire actin structure via 3D confocal z‐stacks. The CZI files were imported, and actin status was analysed using the Zen program software.

### 2.10 Colony formation assay

Cells were seeded in 10 mm plates at a density of 500 cells/plate. After incubation for 10 days at 37°C, the cells were fixed in 4% PFA for 30 minutes and stained with Giemsa stain solution for 20 minutes. The number of colonies consisting of more than 50 cells was counted.

### 2.11 Real‐time cell proliferation and invasion assays

Cell proliferation and invasion abilities were examined using a real‐time cell analysis (RTCA) system (RTCA DP Instrument; ACEA Biosciences, Inc, USA). For continuous monitoring of cell proliferation, the cells were seeded into RTCA E‐plates at a density of 5 × 10^3^ cells per well, and then, the electrical impedance in each well was measured continuously for 50 hours. The shift in electrical impedance is expressed as the cell index, which is a parameter of cell proliferation. The invasion experiments were performed in CIM‐16 plates, and a layer of Matrigel (BD Biosciences) was added to the upper chamber of the plate for an hour. Next, 10 µg/mL of mitomycin was used to inhibit cell proliferation for 3 hours, and then, 3 × 10^4^ cells were seeded and monitored for 80 hours. The sensor impedance following cell invasion was defined as the cell index.

### 2.12 Tube formation assay

Growth factor‐reduced Matrigel was placed in a 96‐well cell culture plate (50 μL/well) and incubated for 30 minutes. EPCR‐KO and control HTR8/SVneo cells (3 × 10^4^/100 μL) were seeded onto the Matrigel‐coated wells. Once tube formation was observed, the medium was aspirated from the wells, and 100 µL of 2 µmol/L calcein AM (Abcam) solution was added to each well of the 96‐well plate. The plate was incubated for 15‐30 min. Tube formation was observed under an inverted microscope. Calcein AM‐labelled cells were photographed using a fluorescent inverted microscope at an excitation wavelength of 488 nm.

### 2.13 MST assay

MST was performed according to a previously described protocol.^22^ In brief, purified recombinant protein was labelled with the RED‐NHS Protein Labeling Kit (NanoTemper Technologies, Munich, Germany) according to the manufacturer's protocol. PAR‐1 protein (Abnova, Taipei City, Taiwan, China) was then incubated at a constant concentration (1 mmol/L) with twofold serial dilutions of EPCR protein (Abnova) in MST‐optimized buffer. Equal volumes of binding reactions were mixed by pipetting and incubated for 15 minutes at room temperature. The mixtures were enclosed in standard‐treated glass capillaries and loaded onto the instrument (Monolith NT.115; NanoTemper). The measurement times were as follows: fluorescence before 5 s, MST at 30 seconds, fluorescence after 5 seconds and delay for 25 seconds. The measurement was performed at 60% MST power.

### 2.14 Cell‐based enzyme‐linked immunosorbent assay

In total, 2 × 10^4^ cells were added to each well of a 96‐well culture plate and incubated overnight. The medium was then removed, and the cells were fixed with 4% PFA and blocked with 1% BSA. The fixed cells were incubated with an anti‐ATAP2 antibody (1:200, Santa Cruz Biotechnology, Dallas, Texas, USA) and an anti‐WEDE15 antibody (1:100, Beckman Coulter, Marseille, France) for 1 hour at room temperature and then washed three times with PBS containing 0.1% BSA followed by incubation with the secondary antibodies for 1 hour. Then, 100 µL of 3,3',5,5'‐tetramethylbenzidine (TMB, Cell Signaling Technology, Inc, Danvers, MA, USA) substrate solution was added to each well and incubated for 20 minutes. The enzymatic reaction was stopped by the addition of 2 M sulphuric acid. The optical density of each well was measured at 450 nm with a multidetector microplate reader (Thermo 1500; Thermo Fisher Scientific, Inc).

### 2.15 Rac1 activation measurement

Cells were grown to confluence in 6‐well culture plates and starved overnight in serum‐free medium. Analysis of Rac1 activation (Rac1‐GTP) was performed using commercially available kits (Cytoskeleton, Inc). Briefly, appropriate cellular lysates were incubated with glutathione S‐transferase‐PAK PBD (Rac1 effector protein, p21‐activated kinase 1) beads that bind specifically to the active GTP‐bound form of Rac1 at 4°C with rotation for 1 hours. The beads were washed and resuspended in loading buffer. Total and activated Rac1 levels were detected by WB analysis using an anti‐Rac1 monoclonal antibody provided in the kit as described by the manufacturer.

### 2.16 F‐actin/G‐actin ratio measurement

The F‐actin/G‐actin ratio was determined using commercially available kits (Cytoskeleton). Briefly, LAS buffer [50 mmol/L PIPES, 50 mmol/L NaCl, 5 mmol/L EGTA, 5 mmol/L MgCl2, 0.1% Nonidet P40, 5% (v/v) glycerol, 0.1% Triton X‐100, 0.1% Tween 20, 0.1% 2‐mercaptoethanol and 0.001% Antifoam C, pH 6.9] was used to lyse the cells. The lysate was centrifuged at 626×*g* for 5 minutes to remove unbroken cells. The supernatant was then centrifuged at 100 000×*g* to separate F‐actin from soluble G‐actin. WB analysis was performed to detect the G‐actin content in the supernatant and the F‐actin content in the sediment.

### 2.17 Statistical analyses

Proliferation and invasion curves were analysed by 2‐way analysis of variance (ANOVA) using SPSS 23.0 software (IBM, Armonk, NY, USA). Student's t test was used for all other data comparisons using GraphPad Prism 6 software (La Jolla, CA, USA). The standard error mean (SEM) was plotted. All experiments were performed three times unless indicated otherwise.

## 3 RESULTS

### 3.1 Trophoblastic EPCR is down‐regulated in preeclamptic placentas

**Figure 1:**
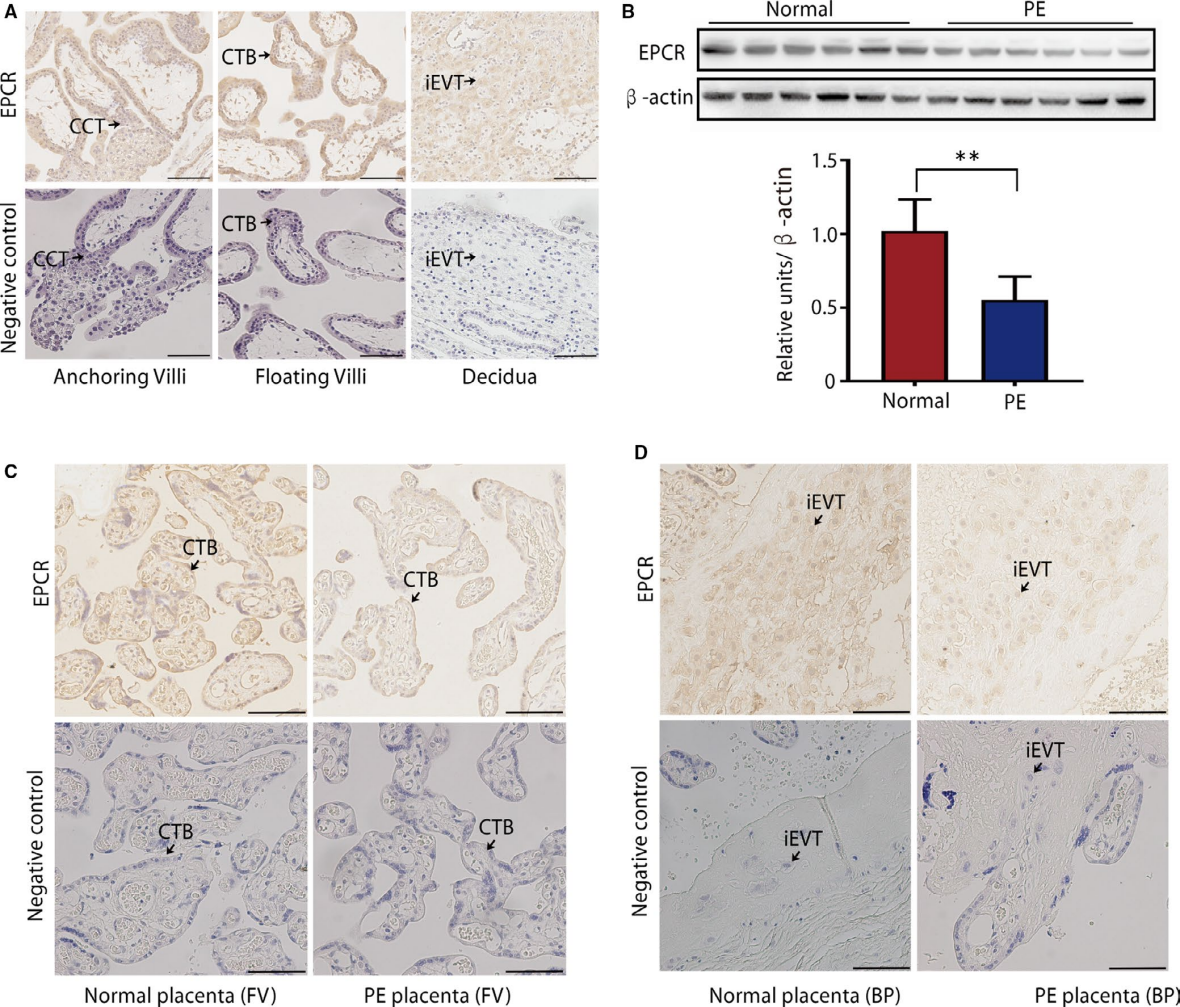
EPCR expression in placentas. (A) IHC staining of EPCR in first‐trimester human placenta villi and the decidua. Scale bar, 100 μm. (B) The expression of EPCR in placental tissues was determined by WB analysis of 6 preeclamptic placentas and 6 healthy control placentas randomly selected from 15 placental tissues. Data are presented as the mean ± standard error of the mean (SEM). **P < .01. (C, D) IHC staining of EPCR in floating villi (C) and BP (D) of control and PE placentas from serial sections. Scale bar, 100 μm

We first used IHC to evaluate the expression levels of EPCR in first‐trimester human placentas. High expression of EPCR was detected in different subtypes of trophoblasts, including villous cytotrophoblasts (CTB), cell column trophoblasts (CCT) and interstitial EVT (iEVT) cells in the decidua (Figure 1A). To further compare the differences in EPCR expression between preeclamptic placentas and placentas from healthy age‐matched subjects, we performed a WB assay to detect the levels of EPCR in both groups. As shown in Table 1, there were no significant differences in maternal age, body mass index, gestational age at delivery or infant birth weight between healthy pregnant (n = 15) and preeclamptic (n = 15) women. The WB results demonstrated that the EPCR level was significantly lower in preeclamptic placentas than in healthy placentas (Figure 1B). The IHC results further revealed strong EPCR staining in the villous CTBs and iEVTs in the basal plate of healthy, preterm placentas, whereas EPCR was weakly expressed in preeclamptic placentas (Figure 1C,D). Collectively, these results showed that the down‐regulation of EPCR in trophoblasts is associated with preeclamptic placentas.

### 3.2 EPCR knockout inhibits trophoblast proliferation and invasion

**Figure 2:**
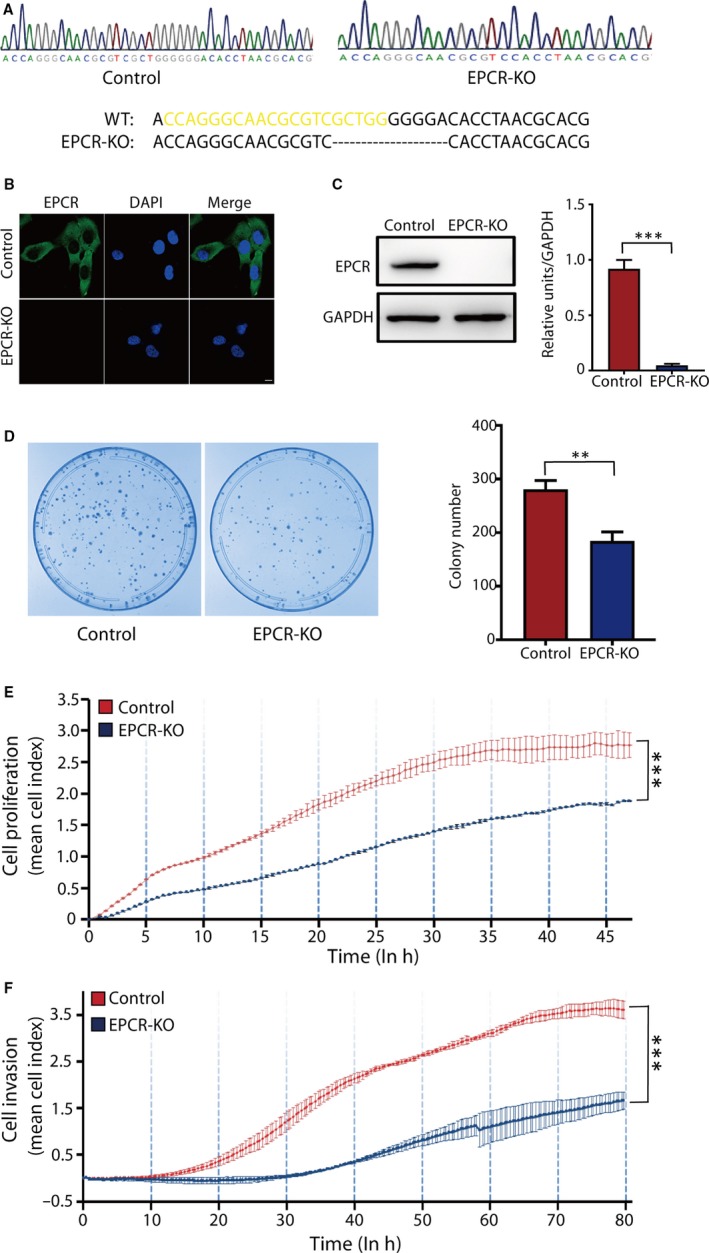
EPCR regulates trophoblast proliferation and invasion. (A) EPCR deletion sequences. Yellow sequences correspond to remains of sgRNA target sequences. (B) Validation of EPCR‐KO by immunofluorescence staining. Green: EPCR; Blue: nucleus. Scale bar, 10 μm. (C) Validation of EPCR‐KO by WB analysis. Equal amounts of control HTR8/SVneo (control) and EPCR‐KO cell lysates were subjected to WB analysis with an EPCR antibody. (D) Colony formation assays of the control and EPCR‐KO groups. The colony formation ability was decreased in the EPCR‐KO group. (E) Proliferation cell index (CI) based on the RTCA real‐time monitoring system. Data were analysed using 2‐way ANOVA. (F) Invasion CI based on the RTCA real‐time monitoring system. Data were analysed using 2‐way ANOVA. All experiments were performed at least three times. Error bars of RTCA curves represent the standard deviation. Other values are represented as mean ± SEM. **P < .01, ***P < .001

To ascertain the causal relationship between trophoblastic EPCR deficiency and PE development, we investigated the role of EPCR in trophoblast proliferation and invasion. We first established a cell line in which EPCR was knocked out using the CRISPR/Cas9 technique on HTR8/SVneo, an EVT‐like first‐trimester trophoblast cell line. First, EPCR deletion bands were sequenced (Figure 2A). The result showed perfect deletion and repair without any indels. Immunostaining and WB results further identified a successful knockout of EPCR in the established cell line (Figure 2B,C). Next, we used colony formation assays to elucidate the role of EPCR in trophoblast proliferation. The results suggested that compared with the control group, the EPCR‐KO group had a significantly decreased number of colonies (Figure 2D). Then, RTCA was performed to further explore the function of EPCR in cell proliferation and invasion. For the proliferation assay, EPCR‐KO and control cells were seeded into E‐plates. At the bottom of the chamber, there are sensors that can detect the number of cells, which are presented as a ‘cell index’. After 50 hours of culture, the cell proliferative ability was significantly decreased in the EPCR‐KO group compared with the control group (Figure 2E). For the invasion assay, a layer of Matrigel was added to the upper chamber of the CIM plates to simulate the extracellular matrix (ECM) and provides a barrier for cell invasion. When the cells passed through the membrane from the upper chamber into the bottom, they contacted the sensors at the membrane and were detected as the cell index.^23^ As shown in Figure 2F, the invasive ability of cells in the EPCR‐KO group was much lower than that in the control group. Thus, these data indicate that EPCR is essential for the proliferation and invasion of trophoblasts.

### 3.3 EPCR knockout inhibits trophoblast tube formation

**Figure 3:**
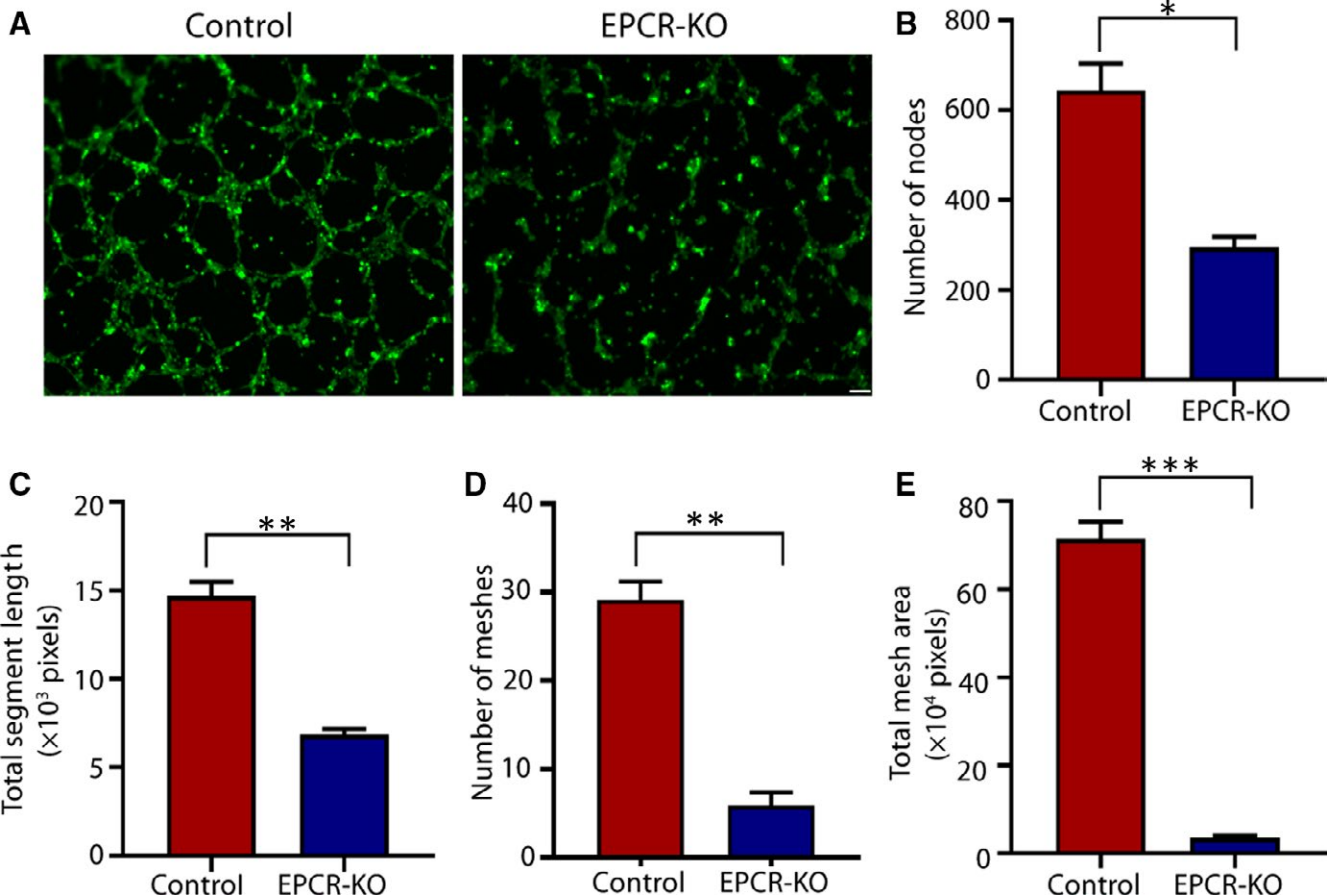
EPCR regulates tube formation in trophoblasts. (A) Matrigel endothelial‐like tube formation was examined in HTR8/SVneo cells. Scale bar, 100 μm. (B‐E) The number of nodes, total segment length, number of meshes and total mesh area was calculated in pixels. All experiments were performed at least three times. All data are represented as the mean ± SEM. *P < .05, **P < .01, ***P < .001

Failure of spiral artery remodelling can result in placental ischaemia and hypoxia and further leads to the occurrence and development of PE.^24^ When the HTR8/SVneo cell line is cultured on Matrigel (with or without endothelial cells), it can spontaneously form endothelial‐like tubes, which is believed to reflect the placental angiogenic ability.^25^, ^26^ To determine whether EPCR expression influences the angiogenic properties of trophoblasts, we performed tube formation assays. As shown in Figure 3A, the EPCR‐KO cells exhibited decreased tube formation compared with the control cells. The quantitative analysis results demonstrated that the number of nodes and meshes, mesh area and total segment length was significantly reduced (Figure 3B‐E). These data demonstrated that EPCR knockout decreases the angiogenic properties of trophoblasts.

### 3.4 EPCR regulates actin polymerization

Actin is crucial in determining cell proliferation and invasion, mainly by F‐actin polymerization and rearrangement of the actin cytoskeleton.^27^ Recent studies suggest that PAR‐1 activation is regulated by EPCR,^28^ which mediates the polymerization of F‐actin and cortical actin distribution.^29^ Therefore, we next investigated whether EPCR knockout affects actin polymerization by activating PAR‐1‐mediated cell signalling.

**Figure 4:**
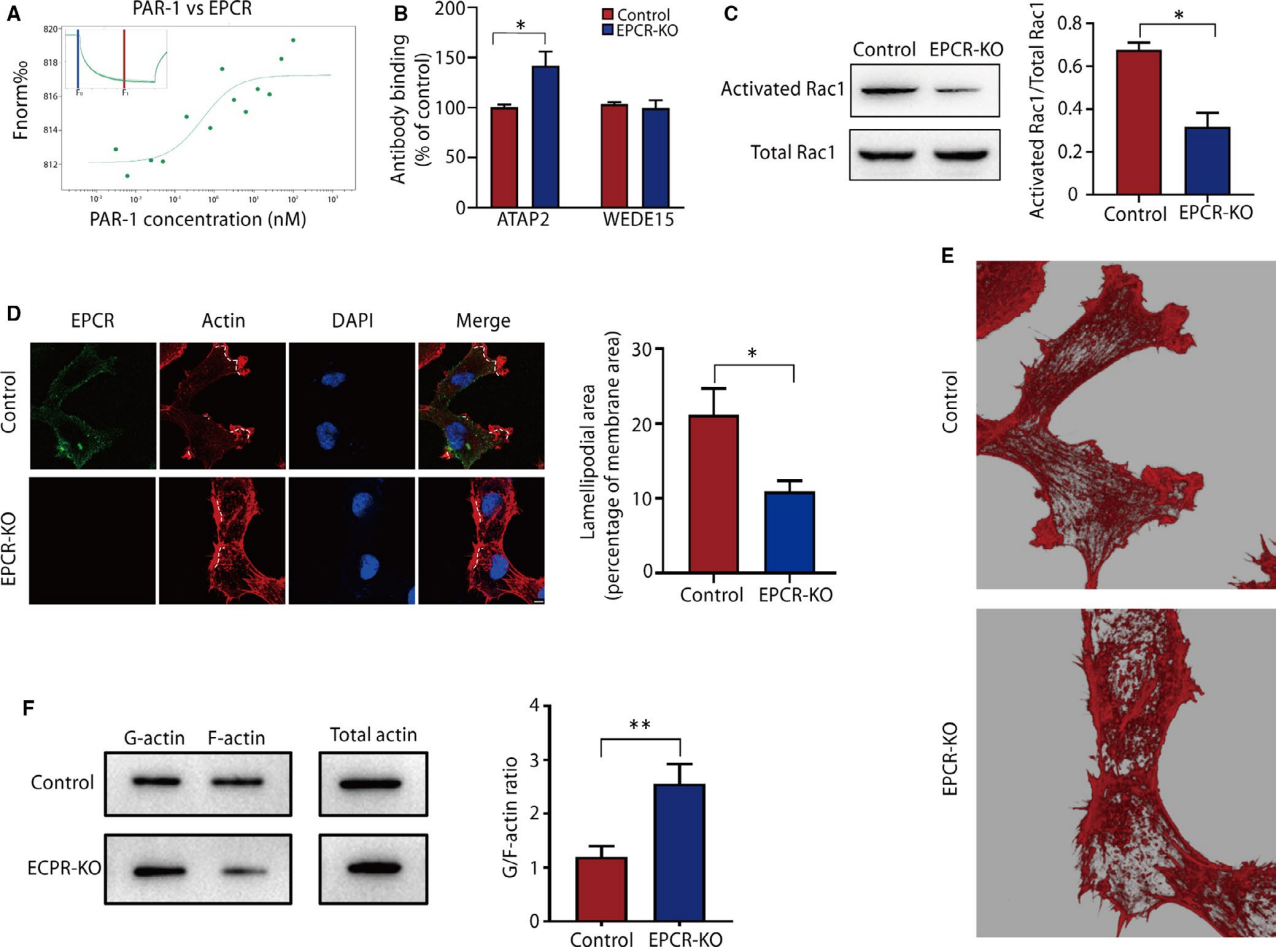
EPCR regulates cytoskeletal rearrangement in trophoblasts by the PAR‐1/Rac1 pathway. (A) MST results of PAR‐1 and EPCR. The change in fluorescence upon switching the laser on and off at 60% intensity is shown. (B) Quantification of cell surface PAR‐1 by a cell‐based ELISA in control HTR‐8/SVneo and EPCR‐KO cells. (C) The levels of total and activated Rac1 were measured by WB analysis. (D) The F‐actin architecture in the EPCR‐KO and control cell groups. Green: EPCR; red: actin; clue: nucleus. Ruffling lamellipodia‐like structures were shown by dotted line. Scale bar, 10 µm. (E) Three‐dimensional modelling of actin in the control and EPCR‐KO cells. (F) G‐actin (soluble), F‐actin (filamentous) and total actin fractions in the control and EPCR‐KO cells were analysed. The results were assessed after normalization to total actin. Data are presented as the mean ± SEM. **P < .01

First, we performed an MST assay to examine the relationship between EPCR and PAR‐1, and we found that EPCR directly bound to PAR‐1 (Figure 4A). Subsequently, the activation of PAR‐1 following EPCR knockout in cells was investigated by a cell‐based enzyme‐linked immunosorbent assay (ELISA). We used a PAR‐1 antibody (ATAP2) that specifically binds to intact PAR‐1 and a non–cleavage‐sensitive antibody (WEDE15) that detects total PAR1 on the cell surface.^30^ We found that the binding rate of the ATAP2 antibody was increased in the EPCR‐KO group compared with the control group, whereas the total PAR‐1 level did not change (Figure 4C). This finding indicated that EPCR knockout inhibited EPCR‐mediated PAR‐1 cleavage.

Cortical actin distribution induced by PAR‐1 is likely mediated by activation of the small Rho family GTPase, Rac1,^31^ which induces rapid actin polymerization in ruffles near the plasma membrane.^32^, ^33^ Next, we evaluated the rate of Rac1 activation in the two groups. As shown in Figure 4C, Rac1 activation was reduced when EPCR was depleted. We also compared the distribution and morphology of actin in EPCR‐KO and control cells through immunostaining analyses. Large accumulation of actin on the membrane surface and formed ruffling lamellipodia‐like structure in the control group. But in the EPCR‐KO cells, actin distribution was disorganized, with few peripheral actin bands. Focal concentrations of actin ‘dots’ or ‘clumps’ were present and randomly distributed. (Figure 4D,E). To support our morphological observations, we assessed the actin cytoskeleton reorganization of EPCR‐KO cells by WB analysis, and the G/F‐actin ratio was determined (Figure 4F). The F‐actin level in EPCR‐KO cells was lower than that in the control group. These data suggest that EPCR knockout results in abnormal actin polymerization.

### 3.5 PAR‐1 activation rescues the abnormal actin organization, decreased proliferation and invasion and inhibited tube formation of EPCR‐KO trophoblastic cells

**Figure 5:**
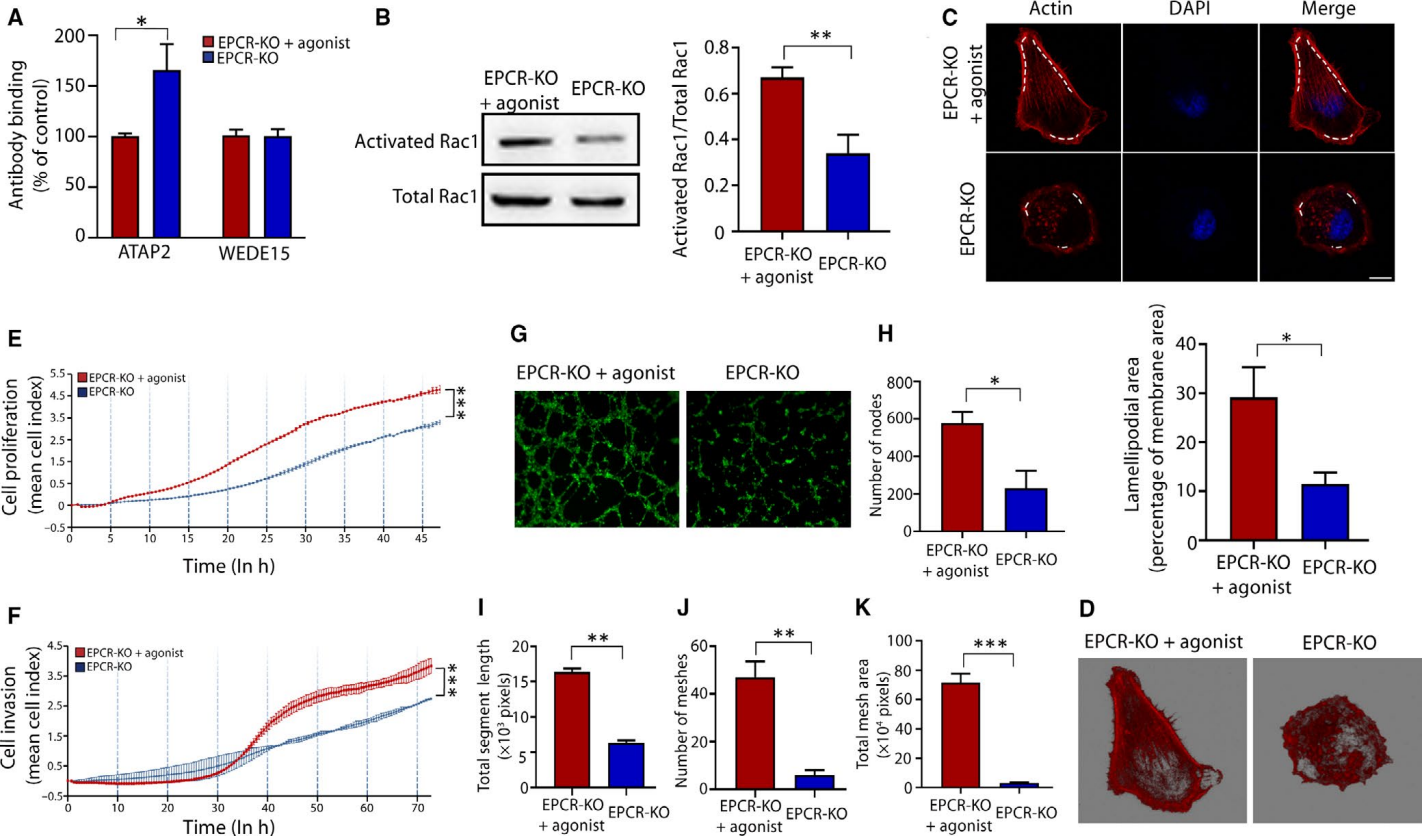
PAR‐1 activation rescues the actin structure and cell behaviour in EPCR‐KO cells. (A) Quantification of cell surface PAR‐1 by a cell‐based ELISA in the EPCR‐KO cells treated by PAR‐1 agonist and EPCR‐KO cells. (B) The levels of total and activated Rac1 were measured by WB analysis. (C) The F‐actin architecture was visualized by rhodamine‐phalloidin staining. Red: actin; blue: nucleus. Ruffling lamellipodia‐like structures were shown by dotted line. Scale bar, 10 µm. (D) Three‐dimensional modelling of actin from each group. (E) Proliferation CI based on the RTCA real‐time monitoring system. Data were analysed using 2‐way ANOVA. (F) Invasion CI based on the RTCA real‐time monitoring system. Data were analysed using 2‐way ANOVA. (G) Matrigel endothelial‐like tube formation in the EPCR‐KO cells treated by PAR‐1 agonist and EPCR‐KO cells. (H‐K) The number of nodes, total segment length, number of meshes and total mesh area was calculated in pixels. All experiments were performed three times. Error bars of RTCA curves represent the standard deviation. Other values are represented as mean ± SEM. *P < .05, **P < .01, ***P < .001

To verify that the effect of EPCR is mediated via PAR‐1/Rac1, we used a PAR‐1 agonist, TFLLR‐NH2, in EPCR‐KO cell lines to validate the effects on actin and cell function. We selected the concentration based on previous reports^34^ and first verified the activation of PAR‐1/Rac1. Using cell‐based ELISAs and WB analysis, we found that the activated PAR‐1 and Rac1 levels were increased after addition of TFLLR‐NH2 (Figure 5A,B).

Then, we explored the effects on actin morphology and cell behaviours. As shown in Figure 5C,D, the actin morphology was restored, and the inhibitory effects on proliferation and invasion of EPCR‐KO cells were reversed (Figure 5E,F). The tube formation ability was also increased (Figure 5G‐K).

### 3.6 Exogenous EPCR rescues the abnormal actin organization, decreased proliferation and invasion and inhibited tube formation of EPCR‐KO trophoblastic cells

**Figure 6:**
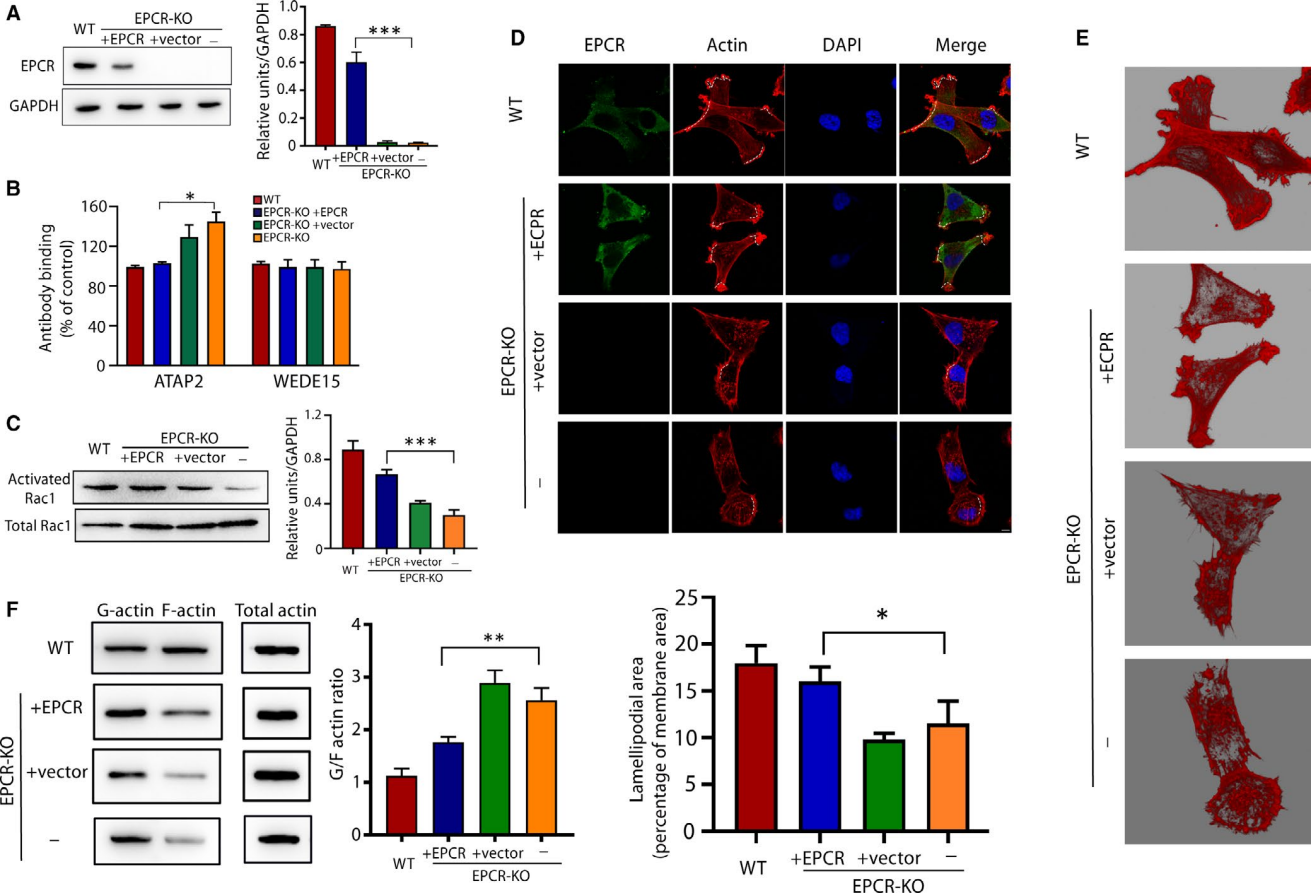
Expression of exogenous EPCR recues F‐actin polymerization. (A) Protein levels of EPCR in the WT, EPCR‐KO and EPCR‐KO cells transfected with the control vector or the EPCR vector. The relative intensity of the bands is summarized in the histogram. (B) Quantification of cell surface PAR‐1 by a cell‐based ELISA in the WT, EPCR‐KO and EPCR‐KO cells transfected with the control vector or the EPCR vector cells. (C) The levels of total and activated Rac1 were measured by WB analysis. (D) The F‐actin architecture was visualized by rhodamine‐phalloidin staining. Green: EPCR; red: actin; blue: nucleus. Ruffling lamellipodia‐like structures were shown by dotted line. Scale bar, 10 µm. (E) Three‐dimensional modelling of actin from each group. (F) G‐actin, F‐actin and total actin fractions in the control, EPCR‐KO and EPCR‐KO cells transfected with the control vector or the EPCR vector were analysed via WB. The data are presented as the mean ± SEM of 3 independent experiments. **P < .01, ***P < .001

To confirm the role of EPCR in actin organization, trophoblast proliferation and invasion and tube formation, we transfected an EPCR‐overexpressing vector into EPCR‐KO cells as a rescue experiment. The WB results confirmed the successful transfection, as the protein level of EPCR was restored in the EPCR‐transfected group (Figure 6A). After transfection, the active PAR‐1 and Rac1 levels were altered (Figure 6B and C), the actin morphology was nearly completely restored (Figure 6D and E), and the F‐actin level was increased to a level similar to that in the wild‐type (WT) cells (Figure 6F).

**Figure 7:**
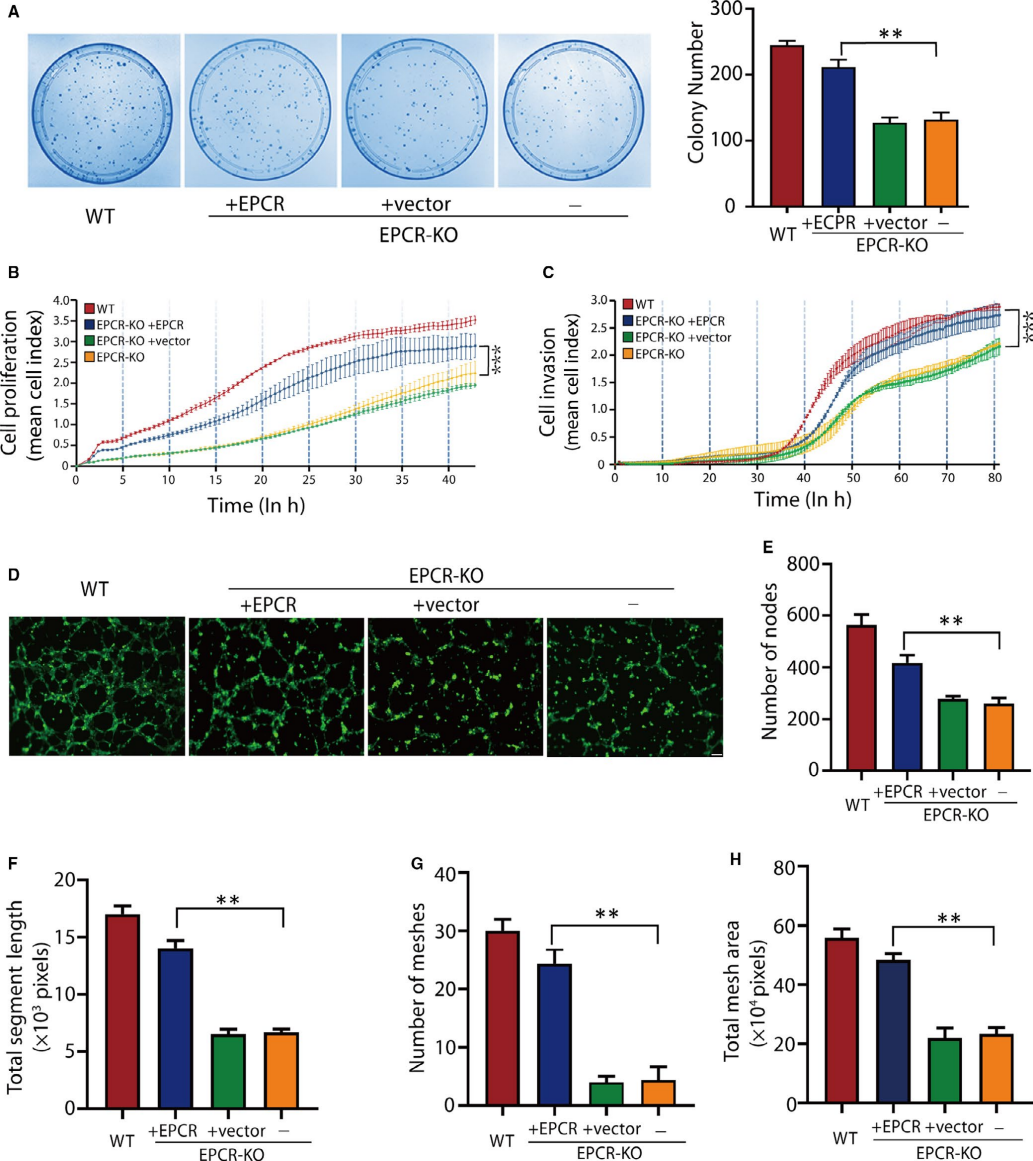
Expression of exogenous EPCR rescues the proliferation, invasion and tube formation abilities of trophoblasts. (A) The colony formation assays of the WT, EPCR‐KO and EPCR‐KO cells transfected with the control vector or the EPCR vector. (B) Proliferation CI based on the RTCA real‐time monitoring system. Data were analysed using 2‐way ANOVA. (C) Invasion CI based on the RTCA real‐time monitoring system. Data were analysed using 2‐way ANOVA. (D) Matrigel endothelial‐like tube formation in the WT, EPCR‐KO and EPCR‐KO cells transfected with the control vector or the EPCR vector. (E‐H) The number of nodes, total segment length, number of meshes and total mesh area was calculated in pixels. All experiments were performed three times. Error bars of RTCA curves represent the standard deviation. Other values are represented as mean ± SEM. *P < .05, **P < .01, ***P < .001

The inhibited proliferation and invasion of EPCR‐KO cells was rescued by exogenous EPCR (Figure 7A‐C). Tube formation was also increased in the exogenous EPCR group (Figure 7D). The data demonstrated that the number of nodes and meshes, mesh area and total segment length was significantly increased (Figure 7E‐H). These results collectively indicate that exogenous EPCR expression can rescue the EPCR knockout phenotype, which further proves that EPCR plays an important role in trophoblastic proliferation, invasion and angiogenesis.

## 4 DISCUSSION

The development of PE can be divided into two stages: in the first stage, the reduced proliferation and shallow invasion of trophoblasts into the endometrium and insufficient spiral artery remodelling cause reduced placental perfusion. In the second stage, impaired placentation induces systemic pathophysiological changes in the maternal circulation. EVTs have been widely reported to play an important role in the first stage of PE.^35^ However, studies on the pathogenesis of trophoblasts in the placenta are insufficient, and the aetiology of PE remains unclear. In the placenta, the depth of infiltration of the trophoblasts should remain moderate, and either exacerbation or failure of trophoblast proliferation and invasion results in pregnancy complications. For example, aggressive invasion is a feature of placental site tumours, such as choriocarcinoma,^36^, ^37^ whereas shallow invasion is characteristic of PE.^35^ Many studies have suggested that trophoblasts are very similar in nature to tumour cells primarily based on the proliferative and invasive properties of these two cell types,^19^, ^20^ and EPCR is highly expressed in various malignancies and is involved in tumour cell proliferation, invasion, metastasis and apoptosis.^38^, ^39^, ^40^ However, the role of EPCR in trophoblasts and PE is not clear. In the present study, we first assessed the expression pattern of EPCR in clinical placenta tissues. In placentas from healthy pregnancies, EPCR was mainly associated with CTBs, and expression was strongly down‐regulated in patients with PE. We then confirmed that the proliferative and invasive activities of trophoblasts were strongly decreased when EPCR was knocked out.

Impaired spiral artery remodelling induced by aberrant EVT endovascular differentiation is believed to contribute to the development of PE.^41^ This change reduces the maternal blood flow into the interstitial space while increasing the blood pressure to levels that are generally incompatible with healthy pregnancy. This phenomenon results in several negative consequences, including disrupted villus trophoblast renewal and necrotic placental debris shedding into the maternal circulation.^42^ HTR8/SVneo cells show endovascular differentiation.^43^ These cells can spontaneously form tubes when cultured on Matrigel ECM and express genes that are associated with actin cytoskeleton organization, cell proliferation and blood vessel development.^44^ In this study, we demonstrated that HTR8/SVneo cells established an endothelial cell‐like capillary network when cultured on Matrigel, but this ability was decreased in EPCR‐KO cells. Together with the expression of EPCR in preeclamptic placentas and its influence on trophoblast proliferation and invasion, these results suggest that EPCR is involved in the progression of PE.

A previous study indicated that EPCR has cytoprotective effects related to cortical cytoskeletal rearrangement via PAR‐1 activation.^45^ PAR‐1 is a G protein‐coupled receptor that mediates signalling through interactions with different members of the G protein subfamilies.^46^ During cytoskeletal rearrangement, actin plays an irreplaceable role through different mechanisms. According to its polymerization and depolymerization status, actin can be divided into filamentous actin (F‐actin) and globular actin (G‐actin).^47^ Although affected by many intercellular and extracellular factors, actin polymerization directly determines cell proliferation and invasion.^7^, ^8^ Rac1 is a critical molecule in the regulation of actin.^48^ This protein regulates various cellular functions through multiple downstream effector pathways by cycling between active and inactive GDP‐bound forms, and the active form of Rac1 can increase F‐actin polymerization.^49^, ^50^, ^51^ Our results showed that depletion of EPCR inhibited the activation of PAR‐1 following inactivation of Rac1. This change caused rearrangement of the actin cytoskeleton and F‐actin depolymerization. Importantly, the overexpression of EPCR in EPCR‐KO cells restored the polymerization of F‐actin, and the proliferation, invasion and tube formation abilities of trophoblasts were enhanced, confirming our hypothesis.

Although our understanding of PE has improved, the mechanisms involved in this pathological condition remain poorly understood. Elucidation of the role of differentially expressed placental‐specific genes in PE patients will enrich our understanding of the pathogenesis of this disease and contribute to its diagnosis and management. We found that EPCR expression was significantly lower in the placentas from PE patients than in those from age‐matched healthy controls, consistent with the finding that EPCR knockout inhibited EVT invasion and proliferation. A previous study^12^ reported that the expression level of EPCR in early‐onset PE was significantly lower than that of the control group, while expression in late‐onset PE showed no significant difference compared with that of the control group. However, in our experiment, the expression levels of EPCR in both early‐onset PE (11 cases) and late‐onset PE (4 cases) were decreased. In addition, IUGR was often accompanied by symptoms of PE. Although the weight of the foetus was generally relatively low in the experimental group, no significant difference in foetal weight between the two groups was found. The expression of EPCR was not significantly different between the samples with and without IUGR in the PE group. These results may be due to the number of cases in our experimental group, especially the number of cases of late‐onset PE, and there were few cases with accompanying IUGR (only 5 samples). We need to expand the sample size to further investigate the expression of EPCR between different preeclampsia subtypes and the relationship with IUGR. A previous study has also reported an increase in plasma soluble EPCR in PE patients.^52^ This may be due to excessive placental ECPR shedding or to other pathways leading to maternal vascular endothelial cell stimulation. Studies have also shown that the expression level of sEPCR is related to gene polymorphisms and specific site mutations.^53^, ^54^ In addition, we did not perform in vivo experiments in this study, and we should further validate our findings in vivo using animal models such as mice.

In summary, for the first time, we elucidated the function of EPCR in the proliferation, invasion and tube formation of human placental trophoblasts and suggested possible pathological mechanisms in PE.

## CONFLICT OF INTEREST

All authors declare that they have no conflict of interest.

## AUTHOR CONTRIBUTIONS

S. Fan designed research; H Wang, P Wang, X Liang, W Li, M Yang, J Ma, W Yue performed the experiments; H Wang and S. Fan wrote the manuscript; and all authors read and approved the final manuscript.

## Data Availability

The data that support the findings of this study are available from the corresponding author upon reasonable request.
